# Perfusion MRI in the evaluation of brain metastases: current practice review and rationale for study of baseline MR perfusion imaging prior to stereotactic radiosurgery (STARBEAM-X)

**DOI:** 10.1259/bjr.20220462

**Published:** 2023-10-24

**Authors:** Caroline Beverley Dobeson, Matthew Birkbeck, Priya Bhatnagar, Julie Hall, Rachel Pearson, Serena West, Philip English, David Butteriss, Joanna Perthen, Joanne Lewis

**Affiliations:** 1 Department of Oncology, Northern Centre for Cancer Care, Freeman Hospital, Newcastle upon Tyne, UK; 2 Northern Medical Physics and Clinical Engineering, Freeman Hospital, Newcastle upon Tyne, UK; 3 Department of Neuroradiology, Royal Victoria Infirmary, Newcastle upon Tyne, UK; 4 Department of Oncology, Northern Centre for Cancer Care, Freeman Hospital, Newcastle upon Tyne, UK; 5 Department of Oncology, Northern Centre for Cancer Care, Freeman Hospital, Newcastle upon Tyne, UK; 6 Department of Neuroradiology, Royal Victoria Infirmary, Newcastle upon Tyne, UK; 7 Department of Neuroradiology, Royal Victoria Infirmary, Newcastle upon Tyne, UK

## Abstract

Stereotactic radiosurgery is an established focal treatment for brain metastases with high local control rates. An important side-effect of stereotactic radiosurgery is the development of radionecrosis. On conventional MR imaging, radionecrosis and tumour progression often have similar appearances, but have contrasting management approaches. Perfusion MR imaging is often used in the post-treatment setting in order to help distinguish between the two, but image interpretation can be fraught with challenges.

Perfusion MR plays an established role in the baseline and post-treatment evaluation of primary brain tumours and a number of studies have concentrated on the value of perfusion imaging in brain metastases. Of the parameters generated, relative cerebral blood volume is the most widely used variable in terms of its clinical value in differentiating between radionecrosis and tumour progression. Although it has been suggested that the relative cerebral blood volume tends to be elevated in active metastatic disease following treatment with radiosurgery, but not with treatment-related changes, the literature available on interpretation of the ratios provided in the context of defining tumour progression is not consistent.

This article aims to provide an overview of the role perfusion MRI plays in the assessment of brain metastases and introduces the rationale for the STARBEAM-X study (Study of assessment of radionecrosis in brain metastases using MR perfusion extra imaging), which will prospectively evaluate baseline perfusion imaging in brain metastases. We hope this will allow insight into the vascular appearance of metastases from different primary sites, and aid in the interpretation of post-treatment perfusion imaging.

## Introduction

Brain metastases reportedly affect between 12 and 40% of patients with metastatic disease from a primary site of malignancy outwith the central nervous system.^1,2^ As systemic treatment options rapidly increase in number and efficacy, with varying degrees of blood–brain barrier penetrance,^3^ increasing numbers of patients are being considered for focal treatment of brain metastases in the context of controlled or controllable extracranial disease.^4^ Focal treatments usually considered are either surgery or ablative radiotherapy.

Stereotactic radiosurgery (SRS) is an established treatment for brain metastases using either a single fraction of high-dose radiotherapy, described as SRS, or a small number of fractions (five or less), described as fractionated SRS (fSRS). SRS is an excellent local treatment for brain metastases with modern data series now suggesting local control rates of treated lesions at 1 year to be 75–90%.^4^

With ever-increasing patient numbers undergoing SRS, experience is evolving in the identification and management of treatment-related radionecrosis (RN). Expertise in interpreting post-treatment imaging is crucial in informing decision-making for these patients and ensuring they are spared from unnecessary interventions.

This article aims to provide an overview of the role perfusion MRI currently plays in the assessment of brain metastases and introduces the rationale for the STARBEAM-X study (Study of assessment of radionecrosis in brain metastases using MR perfusion extra imaging), which looks prospectively at baseline perfusion imaging in brain metastases.

## Radionecrosis

An important observed side-effect of ablative radiotherapy is the development of RN—cerebral parenchymal tissue damage due to radiation. The incidence of RN following SRS is reported to be between 3 and 24% and is typically observed from 6 months up to several years following completion of ablative treatment.^5,6^

RN has complex underlying pathophysiology and current hypotheses of mechanisms of injury are based on both direct damage to glial cells, disruption of the blood–brain barrier and indirect damage to brain parenchyma due to the effect of radiation on the cerebral microvasculature. Histopathological characteristics include necrosis in the white matter, capillary collapse, capillary wall thickening and hyalinisation of the vessels.^6,7^

Clinically, RN can cause neurocognitive impairment, headaches, nausea/vomiting and seizures, but can also be entirely asymptomatic. Radiologically, RN is frequently challenging to differentiate from tumour progression, as its features on imaging—increasing contrast-enhancement, perilesional oedema and mass effect— can mimic that of the latter.^6,8^

The ability to make a diagnosis of RN with confidence is of paramount importance to treating clinicians. Mislabelling appearances on MRI as RN runs the risk of missing true disease progression and, with that, the opportunity to intervene with appropriate management. Equally, incorrectly identifying imaging changes as tumour progression could lead to the unnecessary alteration of a patient’s management with exposure to invasive interventions or systemic treatment changes. Currently, the clinical presentation, timing of onset and imaging findings are interpreted together in order to establish opinion on whether RN or tumour progression is present. Occasionally biopsy is required, although this comes with the not insignificant risks of surgery and does not always yield a definitive diagnosis.^7,9,10^

Factors influencing the development of RN include the treated target volume, location of the treated lesion and radiation dose delivered (including volume receiving 12 Gy (V12Gy) for single fraction treatments.^5,6,10,11^ Much less is understood about host factors in determining risk of RN, however. RN is observed most commonly within the first 24–30 months following SRS,^12^ although the incidence outwith this timeframe is likely to be underreported due to the survival implications of metastatic disease over longer time periods.

RN is primarily treated with systemic corticosteroids in order to reduce the extent of vasogenic oedema. Cases that are steroid-resistant are considered for Bevacizumab,^13^ an anti-VEGF antibody, or surgical resection.^14^

## Imaging

MRI plays a major role in the follow-up of patients with both primary and secondary brain tumours and, as discussed above, has some limitations with regards to differentiating between RN and tumour progression following radiotherapy. Advanced MRI techniques including perfusion, diffusion-weighted imaging, susceptibility-weighted imaging and spectroscopy have come into more frequent use in this field, with perfusion imaging in particular now forming part of the standard investigational approach when the diagnosis is not clear on conventional MRI.^15^

Nuclear medicine imaging has also been at the forefront of research in this area with several studies investigating the use in evaluating post-treatment changes in the brain with radiolabelled tracers such as ^18^F-FDG, ^11^C-MET, ^18^F-FDOPA and ^18^F-FET showing potential value.^16–20^ There are disadvantages, however, of nuclear medicine-based imaging such as access to the appropriate isotopes and the associated radiation dose.

The role of perfusion MRI in assessment of brain metastases has been frequently studied, with recent studies suggesting that MRI biomarkers can predict treatment response from baseline perfusion.^21^ Perfusion MRI provides information about a lesion’s neoangiogenesis and can help inform pre-operative risk assessment and the guidance of stereotactic treatments.^22^

There are three main perfusion MRI techniques to consider: dynamic susceptibility-weighted imaging (DSC), dynamic contrast-enhanced (DCE) and arterial spin labelling (ASL). This review is mainly focussed on DSC imaging as this is the technique to be used in the STARBEAM-X trial, however, a brief introduction to the other techniques and their use in the assessment of metastases is covered. Whilst this review article aims to discuss the outcomes from perfusion MRI data in the assessment of brain metastases, in-depth technical descriptions of each technique are beyond the scope of this article. However, we would like to point the reader to some excellent review articles on various perfusion MRI techniques by authors such as Wintermark et al 2005^23^ and Calamante 2013.^24^

## Dynamic susceptibility imaging (DSC)

The most recognised perfusion MRI technique is DSC-weighted imaging in which serial acquisition of *T*_2_ or *T*_2_* weighted images, following an injection of a paramagnetic gadolinium-based contrast is performed. The arrival of the contrast agent in the brain induces a reduction in the MRI signal on the *T*_2_ and *T*_2_* weighted images due to increased dephasing of the proton spins induced by the change in magnetic susceptibility of blood within the tissue.^25^

From these serial images, various parametric maps can be generated.^26^ The maps are generated by fitting a mathematical function to the image on a voxelwise basis to estimate the time course of the contrast agent. There are three common parameters derived from DSC data; relative cerebral blood volume (rCBV) given by the area under each curve; the relative mean transit time (rMTT) which reflects the time taken from the first signal drop for the signal to return to base; and relative cerebral blood flow (rCBF) which is given by rCBV/rMTT.^22,27^ Other notable parameters include: time-to-peak (TTP), the time taken from the injection of contrast to the lowest signal on the curve and percentage signal recovery (pSR), which is the ratio of the signal during the recirculation phase of the contrast with respect to the baseline signal.

**Figure 1.**
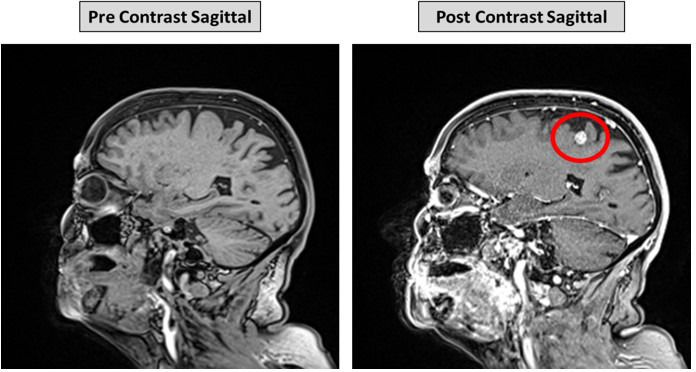
Example pre- and post-contrast images from a 79-year-old female. Evidence of an enhancing metastatic lesion in the right posterior frontal lobe can be seen on the post-contrast image (red circle).

Figure 1 shows example sagittal pre- and post-contrast images from a 79-year-old female at our institution, a mass can be seen in the right posterior frontal region.

**Figure 2.**
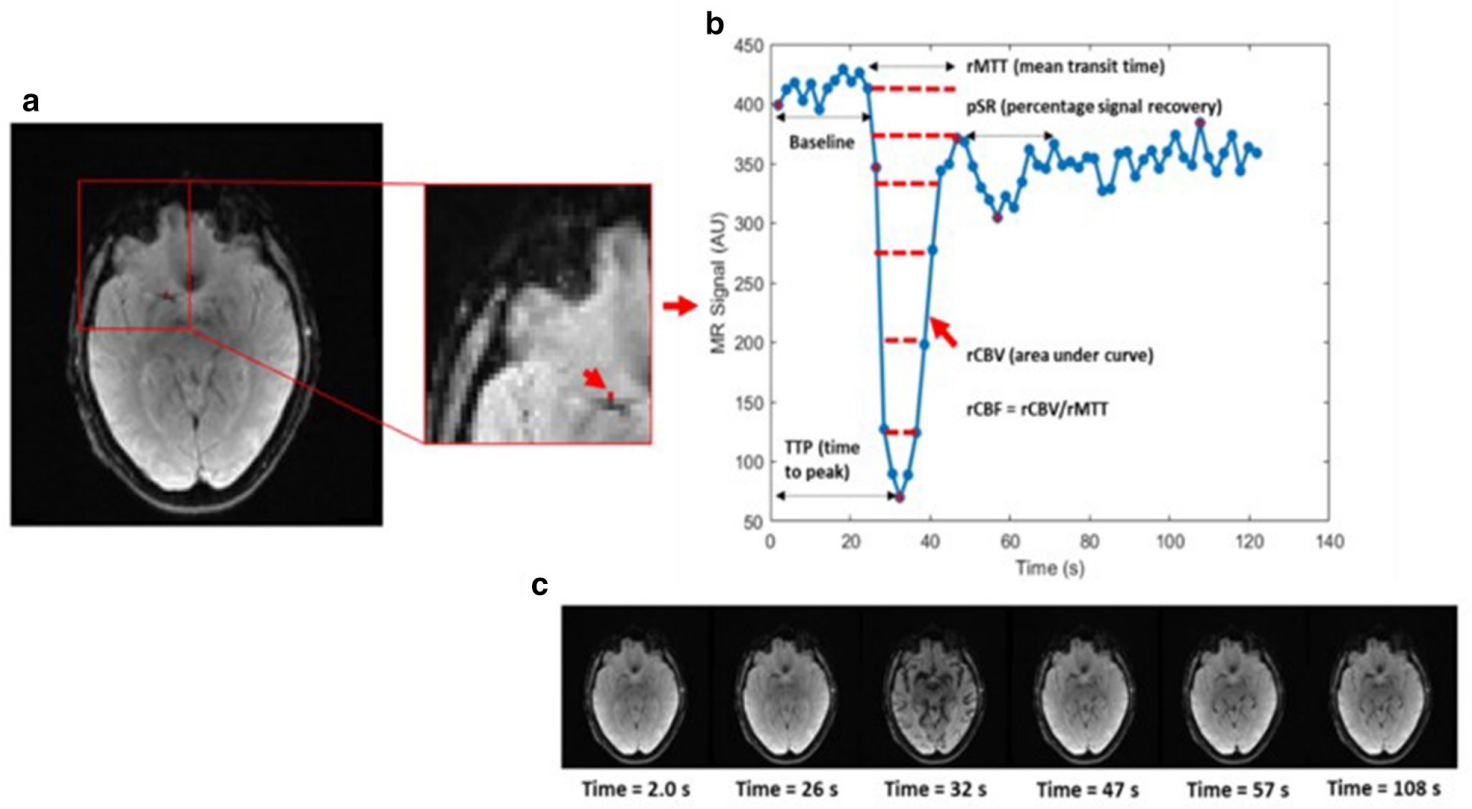
Example DSC data from a 79-year-old female from our institution. (**a**) Left hand image is an axial cross-section of the brain from a DSC perfusion image, with voxel of interest indicated in red. Right hand image is a zoomed in section of the left hand image, voxel indicated by red arrow. (**b**) The time series derived from the red voxel on the image. The parameters are indicated on the time series, indicated by dashed black arrows: Baseline signal, rCBV (area under the curve – indicated by dashed red lines), rMTT, TTP, rCBF = rCBV/rMTT and pSR. (**c**) Example perfusion images corresponding to the time points in (**b**) with red star markers. The images demonstrate the passage of the contrast which can be seen when comparing the baseline image acquired at 2 s with when the contrast arrives into the tissue image acquired at 32 s. DSC, dynamic susceptibility; rCBF, relative cerebral blood flow; rCBV, relative cerebral blood volume; rMTT, relative mean transit time; pSR, percentage signal recovery; TTP, time-to-peak.

Figure 2 shows an example of DSC perfusion imaging acquired in the same patient, but a different area of interest. An example time course from a single voxel is shown with associated time points of the arrival of contrast.

**Figure 3.**
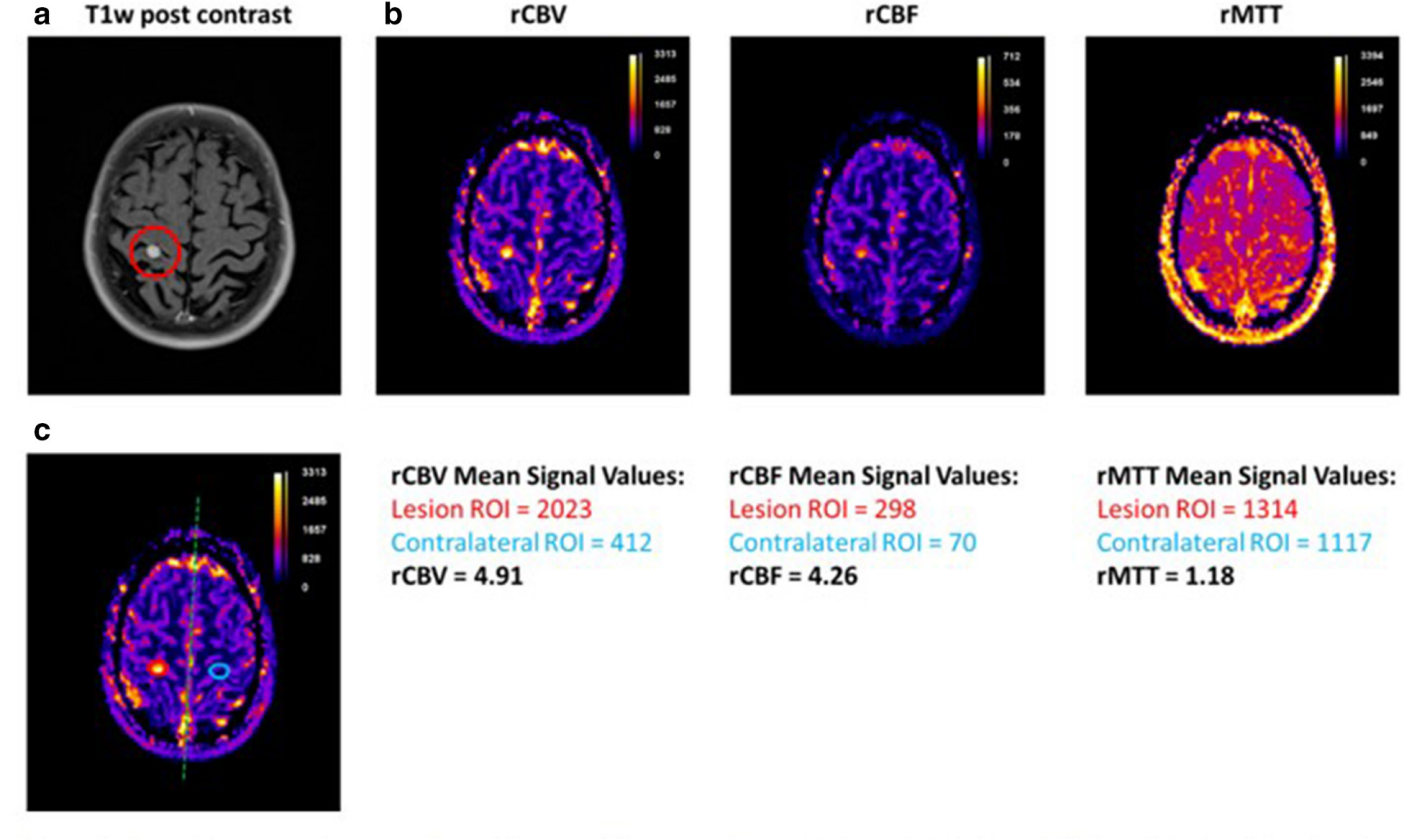
Example parametric maps from the same 79-year-old female at our institution. (**a**) *T*_1_ weighted post-contrast scan demonstrating evidence of an enhancing lesion in the right frontal lobe. (**b**) Parametric maps for rCBV, rCBF and rMTT calculated from the perfusion data, scale bars are indicated on each image. (**c**) Example of how valued are calculated per lesion and then mirrored to the contralateral side of the brain. Calculated values are indicated as ratios for this case for each parameter. rCBF, relative cerebral blood flow; rCBV, relative cerebral blood volume; rMTT, relative mean transit time.

Example parametric maps for the main parameters: rCBV, rCBF and rMTT are shown in Figure 3. Parameters are expressed as relative as they are compared to non-pathological tissue within the brain. This is most commonly performed by drawing a region of interest (ROI) around the lesion and then mirroring this ROI to the contralateral side of the brain. Parameters are then expressed as ratios. Care must be taken when interpreting these ratios as the value is dependent on a number of factors including: the dose of contrast, the software used to process the data, the specific scanner and sequence used to collect the information.^28^

DSC imaging is the most widely used technique to derive perfusion characteristics of brain lesions, with rCBV being the most widely interpreted parameter. It is relatively straightforward to process DSC data, however, the interpretation must be handled with care even between scanners in the same centre. DSC imaging can also be prone to susceptibility artefact either due to calcifications, metal, bone or air interfaces.^28^

## Dynamic contrast-enhanced (DCE) perfusion

This technique similarly involves the application of a contrast agent bolus, but utilises repeated *T*_1_ weighted imaging to observe signal change. This signal change is observed due effect of the contrast agent shortening the T1 relaxation time of tissue.^29^

DCE perfusion imaging allows parametric maps of Ktrans (Volume transfer coefficient between blood plasma and extracellular extravascular space min^−1^) to be generated—this represents the permeability of tumour vasculature and has been shown to be higher in tumour recurrence than in radiation necrosis.^9,30,31^

Compared to the more established DSC, DCE is a relatively new technique. DCE appears to have better spatial resolution and is less susceptible to artefacts, but the images are more complex to acquire and process.^28^

## Arterial spin labelling (ASL)

ASL does not utilise an injected contrast medium, but instead images are generated using magnetically labelled blood as an endogenous contrast agent. ASL can be performed by two techniques; pulsed ASL (pASL) and pulsed continuous ASL (pCASL).^32^

In pCASL, a continuous radiofrequency pulse is applied to magnetically label the inflowing blood to the tissue of interest. The imaging is then performed once this has reached a steady state. This continuous radiofrequency pulse provides excellent perfusion contrast, however, due to the use of a continuous radiofrequency pulse magnetisation transfer effects can cause an overestimation in the perfusion parameters.^28^

In contrast, pASL uses short radiofrequency pulses to magnetically label blood protons most commonly at the neck, this inflowing blood then arrives at the tissue of interest.^33^ ASL allows for direct quantification of perfusion parameters and is particularly useful in patients who have contraindication to contrast agents, paediatric and pregnant patients.^34^

However, due to the lack of contrast agent, the technique has a significantly lower signal-to-noise ratio than counterpart techniques such as DSC or DCE, this leads to longer acquisition times and more potential for movement artefact.^28,32^

Clinically, ASL use has been more frequently used in the assessment of neurodegenerative diseases.^35^

## Clinical application of DSC-MRI in evaluating brain tumours

### Features of malignant lesions on perfusion MR

Perfusion MR has played an established role in the evaluation of primary brain tumours, in particular gliomas, for several years.^36^

Of the parameters generated by DSC-weighted imaging, rCBV is the most frequently studied and widely used variable in terms of its clinical value in differentiating between different conditions affecting the brain. rCBV represents the amount of blood in a given volume of brain tissue (ml/100g of brain tissue) and is calculated by comparing the lesional vascular perfusion compared to that of normal contralateral brain tissue and generating a ratio.^6,37,38^

Studies suggest perfusion appearances of high-grade gliomas typically have increased rCBV, reflecting their increased vascularity and cellular activity compared to normal brain tissue.^39^ The perfusion characteristics across the span of high-grade primary brain lesions also appear to be widely heterogeneous, which is unsurprising in the context of these tumours’ inherent histological characteristics.^40^

Brain metastases from a variety of primary tumour sites have also been assessed using perfusion techniques and compared to primary brain tumours, with studies showing that metastatic lesions tend to have elevated rCBV. Some studies have found that certain parameters measured on MR perfusion sequences can successfully differentiate between hypervascular metastases (*e.g.* melanoma) and hypovascular metastases (*e.g.* lung, breast, colon, renal).^41^ In particular, they found morphological characteristics of the signal intensity–time curve was helpful in differentiating between hyper- and hypovascular metastases. As is described for primary brain tumours, studies report that brain metastases are spatially heterogeneous, and can display different perfusion characteristics in different areas of the tumour (*e.g.* in the core *vs* the rim).^41,42^

### Features of radionecrosis on perfusion MR

A number of studies have concentrated on the value of DSC-MRI perfusion in differentiating RN from tumour progression in treated brain metastases.

One study found that percentage of signal intensity recovery (PSR) values were useful, with PSR >76.3% being indicative of RN with a sensitivity of 95.65% and specificity of 100%.^38^ Other studies, however, have not found statistically significant differences in perfusion values between recurrence and RN, and have reported significant overlap in the values observed in both conditions.^39^

The study team performed a literature search using PubMed and Google Scholar with the following search criteria: ‘brain metastases’, ‘perfusion’, ‘SRS’, ‘stereotactic radiosurgery’, ‘dynamic susceptibility weighted contrast MR’, radionecrosis’. Literature review was performed on results returned over a 15-year period between 1 January 2007 and 1 January 2022. The literature available on interpretation of the ratios provided in the context of defining tumour progression is not consistent. Table 1 provides a summary of the results of relevant published studies, along with rCBV values favouring tumour progression *vs* RN.

**Table 1.** Summary of published studies of perfusion MR in evaluation of brain metastases. DSC - Dynamic susceptibility weighted MR imaging. DCE - Dynamic contrast-enhanced MR imaging. CNS - central nervous system. rCBV - relative cerebral blood volume.

Study	Data collection	MR perfusion technique (Tesla)	Imaging performed (pre-/post-treatment)	Patient number	Lesions studied	Total number of lesions assessed	rCBV threshold for predicting tumour recurrence	rCBV sensitivity	rCBV specificity
Hoefnagels et al^43^	Retrospective	DSC (1.5T)	Post-treatment	31	Brain metastases	34	>2.0 (rCBV-white matter)	85%	71.4%
Morabito et al^30^	Retrospective	DCE/DSC (1.5T)	Post-treatment	28	Primary CNS tumours and brain metastases	72	1.23	88%	75%
Barajas et al^38^	Retrospective	DSC (1.5T)	Post-treatment	27	Brain metastases	30	1.52	91.3%	72.73%
Mitsuya et al**^37^**	Retrospective	DSC (1.5T)	Post-treatment	27	Brain metastases	27	2.1	100%	95.2%
Huang et al^44^	Retrospective	DSC (1.5T)	Post-treatment	26	Brain metastases	33	>2	56%	100%
Wang et al^45^	Prospective	DSC (3T)	Post-treatment	46	Brain metastases	58	2.12	90.9%	96%
Knitter et al^46^	Retrospective	DSC/DCE (1.5/3T)	Pre and post-treatment	29	Brain metastases	32	2.1	75%	50%
Muto et al^47^	Retrospective	DSC (1.5T)	Post-treatment	29	Brain metastases	Not reported	2.1	100%	100%
Kerkhof et al^48^	Retrospective	DSC (1.5T)	Pre and post-treatment	26	Brain metastases	42	Not reported	Not reported	Not reported
Cicone et al^49^	Prospective	DSC (1.5T)	Post-treatment	42	Brain metastases	50	2.14	86.7%	68.2%
Patel et al^50^	Retrospective	DSC (3T)	Post-treatment	23	Brain metastases	25	2.0	75%	79%

CNS, central nervous system; DCE, dynamic contrast-enhanced MR imaging; DSC, dynamic susceptibility-weighted MR imaging; rCBV, relative cerebral blood volume.

It has been suggested that the rCBV tends to be elevated in active metastatic disease following treatment with radiotherapy, but not with treatment-related changes.^34^ One study suggested an rCBV ratio of >2.1 as highly sensitive (100%) and specific (95.2%) for identifying tumour recurrence,^37^ whereas another study from the same year suggested an rCBV cut-off of <0.71 for radiation necrosis with 92% sensitivity and 100% specificity.^35^ Although the literature suggests that there are rCBV value thresholds which can support a diagnosis of either tumour progression or RN with a relatively high degree of certainty, there is clearly an area of overlap where both entities need to be considered.

pSR—an indicator of blood-brain-barrier integrity—reflects the degree of contrast agent leakage through tumour microvasculature and provides insight into the alteration of capillary permeability. This is determined by calculating the percentage of the signal intensity recovery from the lowest signal intensity of the contrast bolus to the end post-contrast signal intensity. One study found that, compared to rCBV, pSR values were the only variable to distinguish between disease recurrence and radiation necrosis with statistical significance.^38^

### Challenges of incorporating perfusion MRI into post-treatment image analysis

The reasons for which perfusion imaging can fail to provide a definitive answer are complex. A hallmark of malignant lesions is their abnormal vascularity and, combined with the inherent heterogeneity of their characteristics even within the same lesion, interpreting the appearance on perfusion imaging to discriminate between two conditions is fraught with challenges. It is also important to recognise tumour recurrence and radionecrosis are not mutually exclusive, and there will be a cohort of patients in which both of these entities are present. The presence of blood products or melanin within a lesion can also cause susceptibility artefacts which complicate perfusion measurements further.^38^

An additional confounding factor is that there is only rarely availability of baseline perfusion characteristics of the metastatic tumour and hence understanding of how results in the post-treatment setting compare to baseline is lost.

Consideration must also be given to the importance of consistency in the processing and interaction of data post-image acquisition. It is critical for the preservation of high-quality results that consistent timing of contrast administration prior to perfusion series is achieved, and that the image processing and data review is performed according to a set protocol and is peer-reviewed.

## Rationale for baseline MR perfusion imaging and development of STARBEAM-X study

As treating clinicians, we are frequently challenged by the diagnostic uncertainty posed by post-SRS imaging appearances raising suspicion of RN or tumour progression. Perfusion MR sequences are a readily available imaging modality that are frequently utilised to aid in decision-making post-SRS. Image acquisition takes little time and costs are relatively low.

Considering the above, our study team propose an approach of assessing brain metastases prior to radiotherapy using baseline MR perfusion imaging as part of the radiotherapy planning process. The rationale for a study of this nature is;

Firstly, the assessment of the vascular appearance of metastases from different primary sites may provide some insight into whether there are any consistent patterns in perfusion appearance according to site of systemic origin. This could be of value in developing future studies focusing on exploiting metastases with certain vascular characteristics therapeutically.

Secondly, we hypothesise that characterising a lesion’s pre-treatment perfusion appearances may aid in the interpretation of subsequent perfusion imaging, with the appreciation of the dynamic changes between pre- and post-treatment time points.

Thirdly, we believe that host factors are likely to influence risk of RN regardless of site of primary malignancy. We therefore aim to study and assess the pre-SRS vascular profile of participants to consider this potential influence on outcome of treatment. To the best of our knowledge, this aspect does not appear to have been studied before when interrogating the available literature.

The STARBEAM-X study aims to recruit 40 participants to undergo baseline MR perfusion imaging prior to treatment with SRS at the Northern Centre for Cancer Care in Newcastle upon Tyne. The study has two phases:

- Phase 1: Recruitment of participants to the study with collection of baseline demographics, cancer diagnosis, baseline MR and perfusion assessment. The host cerebrovascular environment will be assessed and each metastasis will be evaluated on perfusion sequences.
- Phase 2: 24-month period of observation of participants with data recorded on local control following SRS, identification of participants with suspected RN and outcome of repeat MR perfusion assessment.

To participate in the study, patients must have one or more intact brain metastases from an extracranial primary solid malignancy and must not have received any cranial radiotherapy prior to the baseline perfusion MRI. Baseline and post-treatment rCBV values will be compared and the change between the values will be reported, including the influence of these values on clinical decision-making and diagnostic certainty. A specific rCBV threshold will not be used in order to diagnose RN; the study team hypothesise that the change between rCBV values on pre- and post-treatment imaging may be more clinically useful.

At our study site, baseline perfusion MRI has been performed on three patients to assess feasibility. Perfusion data were acquired on a Siemens Magnetom Sola 1.5T scanner. Scan parameters were: field of view = 230×230 mm, in-plane resolution = 1.8×1.8 mm, slice thickness = 5 mm, TE/TR = 31/1920 ms. Scans were acquired during a bolus injection of gadovist contrast agent over 60 phases. The MRI protocol including pre- and post-contrast sequences was 30 min in duration. rCBV perfusion maps were calculated in Syngo.Via software and interpretation performed by an expert neuroradiologist. Example perfusion maps from one patient are shown in Figure 3. rCBV was successfully extracted from the maps in all three patients. The addition of the perfusion MRI sequence added 2 min to the existing MR protocol.

Image quality was sufficient for accurate clinical diagnosis at 1.5T. Perfusion data were of sufficient quality to assess baseline perfusion characteristics of tumours in all patients (See example in Figure 3).

The study team look forward to presenting the results of STARBEAM-X following completion—the study team recognise the intended recruitment target of 40 patients is yield a relatively small number of cases of radionecrosis. It is our hope, following this initial study, to expand baseline perfusion imaging within a study of a larger group of participants.
